# Hydrothermal Conversion of Giant Reed to Furfural and Levulinic Acid: Optimization of the Process under Microwave Irradiation and Investigation of Distinctive Agronomic Parameters

**DOI:** 10.3390/molecules201219760

**Published:** 2015-11-30

**Authors:** Claudia Antonetti, Enrico Bonari, Domenico Licursi, Nicoletta Nassi o Di Nasso, Anna Maria Raspolli Galletti

**Affiliations:** 1 Department of Chemistry and Industrial Chemistry, University of Pisa, Via Giuseppe Moruzzi 13, Pisa 56124, Italy; claudia.antonetti@unipi.it (C.A.); domenico.licursi@virgilio.it (D.L.); 2 Land Lab, Institute of Life Sciences, Scuola Superiore Sant’Anna, P.za Martiri della Libertà 33, Pisa 56127, Italy; enrico.bonari@gmail.com (E.B.); n.nassiodinasso@sssup.it (N.D.N.)

**Keywords:** hydrothermal process, giant reed, furfural, levulinic acid, dilute hydrochloric acid, homogeneous catalysis, microwave

## Abstract

The hydrothermal conversion of giant reed (*Arundo donax* L.) to furfural (FA) and levulinic acid (LA) was investigated in the presence of dilute hydrochloric acid. FA and LA yields were improved by univariate optimization of the main reaction parameters: concentration of the acid catalyst, solid/liquid ratio of the reaction mixture, hydrolysis temperature, and reaction time. The catalytic performances were investigated adopting the efficient microwave (MW) irradiation, allowing significant energy and time savings. The best FA and LA yields were further confirmed using a traditionally heated autoclave reactor, giving very high results, when compared with the literature. Hydrolysis temperature and time were the main reaction variables to be carefully optimized: FA formation needed milder reaction conditions, while LA more severe ones. The effect of the crop management (e.g., harvest time) on FA/LA production was discussed, revealing that harvest time was not a discriminating parameter for the further optimization of both FA and LA production, due to the very high productivity of the giant reed throughout the year. The promising results demonstrate that giant reed represents a very interesting candidate for a very high contemporary production of FA and LA of up to about 70% and 90% of the theoretical yields, respectively.

## 1. Introduction

**Scheme 1:**
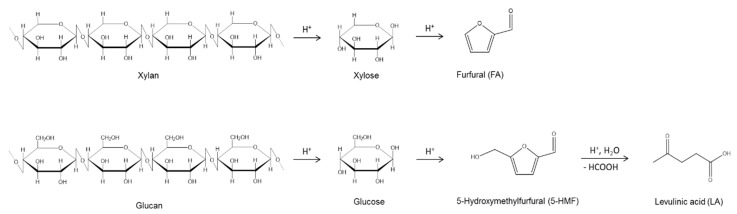
Synthesis of Furfural (FA) and Levulinic acid (LA) in the presence of an acid catalyst.

The hydrothermal conversion of biomass represents a sustainable solution to the increasing demand of bio-fuels and bio-chemicals, allowing security of supply and economic advantages, especially when cheap raw materials are employed as feedstock [1,2,3]. In this context, the selective synthesis of furfural (FA) and levulinic acid (LA) by means of acid hydrothermal conversion of hemicellulose and cellulose biomass fractions has gained increasing attention, due to the many upgrading possibilities of both these platform chemicals. In fact, FA and LA are valuable and versatile precursors for the production of different chemicals, such as alternative fuels, fuel additives, solvents, dyes, flavoring agents, and various resins [4,5,6,7,8,9,10,11,12,13,14,15,16,17,18,19,20,21,22]. In particular, furfural is the key platform for both the chemical and fuel industries. It can replace the diminishing fossil-based organics for the preparation of resins, lubricants, adhesives, and plastics. It is also widely employed to obtain value-added products, such as furfuryl alcohol, tetrahydrofurfuryl alcohol, furanoic acid, and tetrahydrofuran. Levulinic acid is a renewable platform molecule which has also been identified as one of the United States Department of Energy’s (DOE’s) top 12 value-added biochemical [23], finding applications for several purposes, such as a source of polymer resins, animal feed, food, as well as components of flavoring, the fragrance industry, textile dyes, additives, extenders for fuels, antifreeze products, antimicrobial agents, herbicides, and also plasticizers. Nowadays, FA is mainly produced by dehydration/hydrolysis of pentoses [5,10,11,24,25], whilst LA derives from the degradation of hexoses [10,20,26,27,28,29], both reactions being catalyzed by the presence of acid systems, according to Scheme 1:

The effectiveness of these acids depends on their concentration, strength of their primary dissociation constants, and type of feedstock. The catalytic conversion of xylose, xylan, glucose, glucan, and pre-treated biomass substrates into FA and LA by means of monophasic and biphasic solvents has been widely investigated by using homogeneous and heterogeneous catalytic materials containing Lewis and Brønsted functionalities [30]. In this context, homogeneous mineral acids, such as HCl or H_2_SO_4_, have been extensively investigated and applied for FA and LA production on an industrial scale. In particular, the high activity of hydrochloric acid is well-known [31,32,33]. In fact, the use of HCl, instead of H_2_SO_4_, avoids the salt precipitation and limits the deposition of humin by-products, thus minimizing the possibility of reactor clogging and maximizing at the same time the LA yield [1]. Furthermore, the use of hydrochloric acid is clearly advantageous thanks to its high volatility, e.g., easy recovery by atmospheric/vacuum distillation and steam stripping, allowing ∼95% of the acid catalyst and water to be recycled after the cascade process for FA and LA production [3,34].

The choice of the appropriate biomass source for the synthesis of these bio-products is strategic from both techno- and socio-economical points of view. Usually, waste streams with a low or even negative value, such as agricultural wastes, are preferred, because their employment for chemical transformations could represent a way to exploit wastes, producing additional incomes for farmers and limiting the land use competition for food production. On the other hand, the use of perennial rhizomatous species, such as giant reed (*Arundo donax* L.), in abandoned or marginal lands is receiving increasing attention as possible feedstock to be exploited by hydrothermal processes. Their introduction in the convectional cropping systems could be not only an innovative opportunity for farmers but also a source of several environmental advantages (e.g., reduction of GHG emissions, control of soil erosion, remediation of soil *etc.*) [35,36,37,38].

In particular, several distinctive traits contribute to justify the exploitation of giant reed for the production of renewable products, such as its rapid growth rate, its high resource-use efficiency (regarding water, radiation and nutrients), its tolerance of biotic (pests and diseases) and abiotic (heat, freezing, salt *etc.*) stress, and its relatively low production costs [39,40]. In the Mediterranean area, giant reed yields ranged between very high values (over 35 Mg·ha^−1^·year^−1^) on fertile soil to lower values (around 20 Mg·ha^−1^·year^−1^) in marginal lands [40,41]. In the same environment, the analysis of the giant reed growth highlighted that its yield usually peaks in autumn and is almost steady in winter [42]. Monti *et al.* [43] have recently confirmed that there is a connection between harvest time and biomass productivity/quality (moisture content, ashes, cellulose, and hemicellulose). In general, climate can influence the characteristics of the biomass and harvest time could be considered as a key point to direct giant reed crops towards different supply chains since its optimization strongly affects the sustainability of the whole chain. In general, two harvest times were suggested for the thermochemical conversion of giant reed, autumn and winter, while a larger harvest time window can be adopted when giant reed biomass is addressed to biological and chemical conversion including also double harvest systems (e.g., bioethanol and biogas production) [44,45].

From an industrial chemical point of view, giant reed biomass is of great interest, because of its high content of sugar macro-constituents, which are cellulose and hemicellulose [46,47]. These require different reaction conditions for their solubilization/conversion and, in particular, the effects of kind/concentration of the acid catalyst, hydrolysis temperature, and reaction time have to be individually investigated and optimized. FA and LA yield can be reported on a ponderal basis (*i.e.*, weight % with respect to the starting biomass) but, even better, with respect to the maximum theoretical yield. In this last way, FA and LA yield is evaluated both with respect to the hemicellulose/cellulose content and to the stoichiometry of the hydrolysis reaction, because these macro-constituents are also converted to by-products. On this basis, the maximum theoretical yields of LA from hexoses and cellulose are 64.5 wt % and 71.5 wt %, respectively, due to the co-formation of formic acid, whilst that of FA from pentoses amounts to 72.7 wt %. An ideal biomass should have the highest content of cellulose/hemicellulose and the lowest one of lignin. The first requisite is necessary for maximizing the yield of LA and FA, while the second one is important to have a lower resistance (recalcitrance) to the hydrolysis, which should require milder reaction conditions for the conversion to FA and LA and generate a lower amount of residues.

Microwave (MW) irradiation represents a very appropriate tool to make the hydrothermal process more efficient. This form of energy can interact very efficiently with polar molecules, thus allowing a rapid heating of the reaction environment and better yields and selectivity towards the desired products. MW radiation can penetrate lignocellulosic materials and the heat can be produced throughout the volume of the materials (in core volumetric heating) rather than an external source. Furthermore, in the case of lignocellulosic biomass, the effect of MW irradiation at a molecular level leads to a physical disruption of the internal composition of cells, increasing the rate of mass transfer. The application of microwave heating is a fast growing research area, where high reaction rates and selectivity can be achieved together with a significant reduction of the reaction time (often by orders of magnitude) and of energy consumption [48,49,50,51]. In addition, reproducible experiments can be carried out, especially thanks to the use of “mono-mode” (also named “single-mode”) MW applicators. Hydrothermal biomass conversion exploits water as green solvent to be used at high temperature and pressure [52]. At room temperature, water has a high dielectric constant ε’ and an intermediate loss tangent (tan δ), thus being classified as a medium MW-adsorbing solvent. Besides, it represents a strategic choice for biomass deconstruction because some of its properties, such as dielectric constant, density, and ionic product, can be adjusted with the temperature and pressure [53]. Especially hot temperature water (HTW), alternatively named subcritical liquid water (150 °C < T < 374 °C), becomes less dense and the dielectric constant and the level of hydrogen bonding decrease in the temperature range of 180–300 °C, if compared to water at room temperature. As a result, HTW behaves like many organic solvents with a high solubility for biomass-derived molecules. In addition, HTW becomes a stronger electrolyte by dissociation, resulting in higher concentrations of H^+^ and OH^−^ that can catalyze chemical reactions, such as carbohydrate dehydration and hydrolysis routes [54], at the same time allowing high solubility and homogeneous dispersion of the intermediates in the whole solution [55]. From this new perspective, the use of microwave irradiation for the investigation of the hydrolysis reaction represents a versatile choice, being used both for the selective milder hydrolysis of C5 and C6 polysaccharides to solutions rich in simpler monosaccharides or to furanic intermediates (e.g., FA and 5-HMF, respectively) and for the harsher hydrolysis of intermediates deriving from carbohydrate conversion into LA. When raw biomass, such as wheat straw, is used as substrate, FA is the major product obtained using HCl in a MW-assisted process [56]. In our recent work [57], the MW-assisted hydrothermal conversion of giant reed and switchgrass into FA was preliminarily investigated, by using quite dilute hydrochloric acid and mild reaction conditions, thus approaching a very good FA yield. Gómez Bernal *et al.* [58] recently investigated the FA synthesis starting from corn stover hemicelluloses, by using a MW-assisted, green and heterogeneously catalyzed two-step cascade process, as follows: first step, hydrothermal fractionation of corn stover hemicelluloses, and second step, hydrolysis/dehydration of soluble hemicellulosic sugars over niobium phosphate to yield FA at moderate temperatures (<200 °C), with both steps being performed in water.

Regarding LA synthesis, a combined ultrasound/MW assisted device was applied in the acid-catalyzed hydrolysis of corn starch [59]. Non-edible clean biomasses, such as chitosan or cellulose, were used by Szabolcs *et al.* [34] as substrates for MW-assisted LA production in the presence of dilute mineral acids. Rivas *et al.* [60] recently investigated the production of LA starting from HCl-catalyzed hydrothermal treatment of *Pinus pinaster* wood in a multi-mode MW reactor. In our previous work [1], the homogeneous acid-catalyzed hydrothermal conversion of waste biomasses (poplar sawdust, paper mill sludge, tobacco chops, wheat straw, olive tree prunings) was studied and it was found that MW irradiation was efficient for the heating of the aqueous slurry, thus maximizing LA yields (depending on the starting feedstock). More recently, Tabasso *et al.* [61] recently studied and optimized the MW-assisted conversion of a tomato plant waste into LA, achieving a good LA yield.

Starting from all the above results, the aim of this work was the optimization of the giant reed (*Arundo donax* L.) hydrothermal conversion into FA and LA by means of a MW approach. In detail, in the first part of the work, the MW-assisted dependence of the main reaction parameters both on FA and LA production was investigated. The reaction parameters include the concentration of the acid catalyst, the solid/liquid ratio of the reaction mixture, the hydrolysis temperature, and the reaction time. Hydrochloric acid was used for this catalytic investigation, due to its previously mentioned advantageous properties. In order to complete this first part of the work, the giant reed hydrolysis reaction to give FA and LA was also studied in a traditionally heated autoclave reactor.

Few studies regarding the dependence of FA/LA yield from the harvest time of biomass have been carried out up to now [62], and generally the agronomic parameters of the studied biomass have not been considered and reported. Therefore, in the hydrothermal process for FA and LA production, it was necessary to perform an integrated chemical-agronomic study, which represents a valuable issue to pursue in order to achieve maximum economic optimization. In the second part of this work, an integrated chemical-agronomic study of giant reed conversion into FA and LA was carried out, in order to evaluate the giant reed exploitation on a larger application scale, such as that of an integrated biorefinery, taking into account some of its agronomic characteristics, up to now only scarcely highlighted in most literature papers. In this sense, the crop productivity at different harvest time was analyzed and these data related to those of the best FA and LA yields.

## 2. Results and Discussion

### 2.1. Optimization of the Biomass Hydrolysis Conversion to Give Furfural and Levulinic Acid

**Scheme 2:**
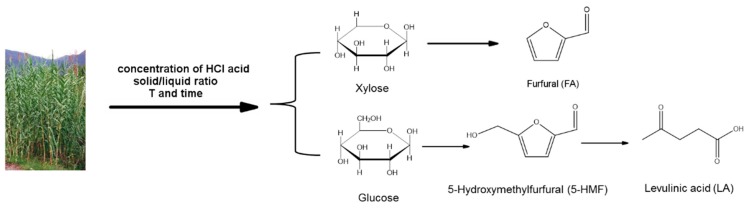
Synthesis of Furfural (FA) and Levulinic acid (LA) in the presence of HCl catalyst from giant reed.

Preliminary hydrolysis tests were performed on many giant reed samples, in order to study the effect of the main reaction parameters on FA and LA yields, according to the reaction mechanism reported in Scheme 2.

In detail, solid/liquid ratio of the reaction mixture, hydrochloric acid concentration, hydrolysis temperature, and reaction time were changed, by adopting an univariate approach. Especially in the case of FA synthesis, hydrolysis temperature and time have a significant influence and therefore these reaction parameters were fully investigated. In fact, it is well known that long reaction times and high temperatures cause FA degradation in the liquid phase. On the other hand, LA formation requires more harsh reaction conditions (temperature and time). Also in the case of LA synthesis, temperature and time are the most important reaction parameters, but LA optimization is easier because this bio-product is obtained under thermodynamic control.

Regarding the concentration of the acid catalyst, this was employed at a very dilute concentration, in order to minimize the problems of plant corrosion and ensure process sustainability. On this basis, different concentrations of hydrochloric acid were tested, never exceeding ~2 wt %. When very dilute hydrochloric acid concentrations, short reaction times, and high temperatures were used, the hydrothermal process could still be considered as a mild one. However, in order to simplify the univariate approach, the same concentration of HCl was adopted in the hydrolysis reactions for the production of FA and LA, respectively.

**Figure 1:**
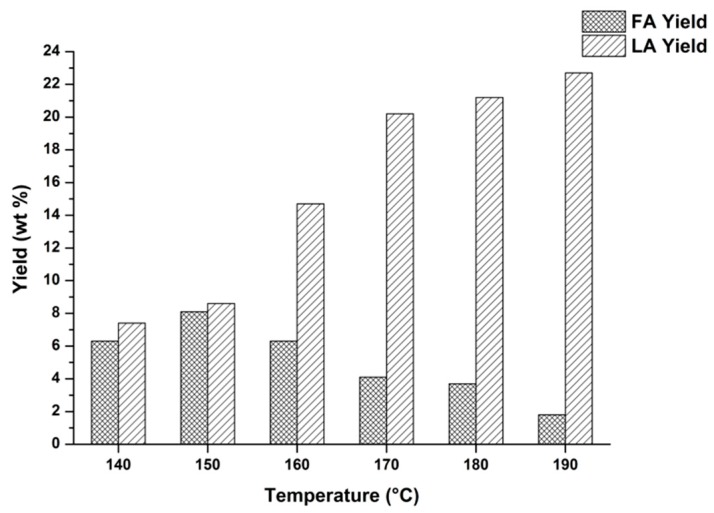
Optimization of giant reed hydrolysis in a MW reactor: yield in furfural (FA) and levulinic acid (LA) as function of temperature. Agronomic data: “*Pisa*” ecotype; harvest time: October; crop age: 4 years. Operating conditions for FA synthesis: 0.35 g giant reed, 5.00 g water, HCl concentration: 1.68 wt %. Hydrolysis time: 15 min. Operating conditions for LA synthesis: 1.63 g giant reed, 23.33 g water, HCl concentration: 1.68 wt %. Hydrolysis time: 20 min.

Regarding the choice of solid/liquid ratio, biomass hydrolysis proceeds under heterogeneous reaction conditions, because of its high recalcitrance to be hydrolyzed. Therefore, it was not possible to enhance too much the solid/liquid ratio of the reaction mixture. On this basis, temperature and time were the main reaction parameters which were investigated by the MW approach. The effect of MW irradiation on biomass hydrolysis was advantageous mainly by adopting short reaction times and therefore the window of the investigated reaction times was narrow and limited, never exceeding one hour. Temperature represented the main parameter of the process to be optimized, this choice being further supported by the perfect control of this parameter with the MW instrumentation [63]. In order to identify the appropriate reaction temperatures for the FA and LA synthesis, a wide screening of the reaction temperatures was studied, e.g., between 130 and 190 °C and the obtained hydrolyzates were analyzed by GC-MS (see Supplementary Information, Figures S1 and S2). On the basis of this preliminary quick investigation, it was found that FA was the main product of hydrolysis at lower temperatures (up to 160 °C), whereas LA was mainly obtained at higher temperatures (above 160 °C). Subsequently, these hydrolyzates were further analyzed by UV-Vis and GC for FA and LA quantification, respectively. The results are reported in Figure 1 for the selected giant reed sample.

**Figure 2:**
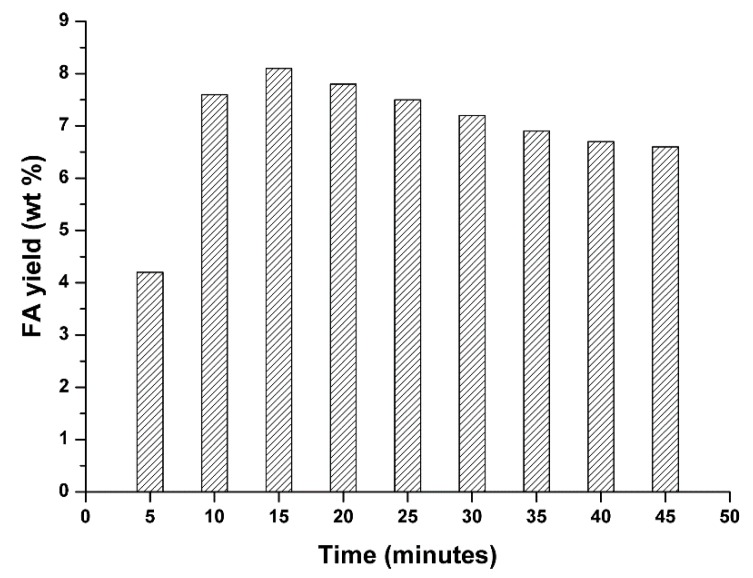
Optimization of giant reed hydrolysis in a MW reactor: yield in furfural (FA) as function of time. Agronomic data: “*Pisa*” ecotype; harvest time: October; crop age: 4 years. Operating conditions: 0.35 g giant reed, 5.00 g water, HCl concentration: 1.68 wt %. Hydrolysis temperature: 150 °C.

The above results show that the FA and LA yields markedly depend on reaction temperature. In particular, FA can be recovered in high yields (~8–9 wt %) from the reaction mixture by using quite mild reaction conditions, which are a low hydrolysis temperature, up to 150 °C and a short hydrolysis time, 15 min. Under the optimal reaction conditions for FA synthesis, a blank test in the absence of HCl catalyst was also carried out and, in this case, only trace amounts of FA were obtained, thus confirming the effectiveness of our catalytic approach. FA is an intermediate compound, obtained under kinetic control. The use of more severe reaction conditions (*i.e.*, higher acid concentration and/or reaction temperature/time) causes FA degradation in the liquid phase, due to competitive FA condensation/resinification reactions [64,65], leading to a progressive worsening of FA yield. This statement is further confirmed in Figure 2, where the dependence of FA yield on the reaction time is reported for the selected giant reed sample.

The high FA yields evidenced in Figure 2 were favored by the fast heating and cooling protocol under the microwave irradiation procedure, which can be reproduced only with greater difficulty under conventional heating protocols.

The severe reaction conditions which lead to FA degradation, e.g., higher hydrolysis temperatures (170–190 °C) and longer hydrolysis times (20 min), are appropriate for LA synthesis, giving a maximum yield in this bio-product equal to about 21–23 wt %, under thermodynamic control. Therefore, the reaction time seemed less important than the temperature for LA optimization, as confirmed by the LA data reported in Table 1:

**Table 1:** Optimization of giant reed hydrolysis in a microwave (MW) reactor: yield in levulinic acid (LA) as function of time and temperature. Agronomic data: “*Pisa*” ecotype; harvest time: October; crop age: 4 years. Operating conditions: 0.35 g giant reed, 5.00 g water, HCl concentration: 168 wt %.

Reaction Time (min)	Temperature (°C)	LA Yield (wt %)
20	180	21.1
15	180	20.2
10	180	15.2
20	140	7.4
15	140	5.1
10	140	Traces

The above data confirm that temperature is the main parameter to be optimized by the MW approach, whilst the gain obtained by the time optimization is rather modest, beyond a certain determined limit. Also in this case, the blank experiments in the absence of HCl catalyst carried out at the same optimal temperatures for LA production in the presence of the acid (180 and 190 °C on the basis of Figure 1 and Table 1) gave very low LA yields (about 1 wt %), thus further confirming that dilute hydrochloric acid represents a very efficient catalytic system for the conversion of giant reed into these valuable platform chemicals.

**Figure 3:**
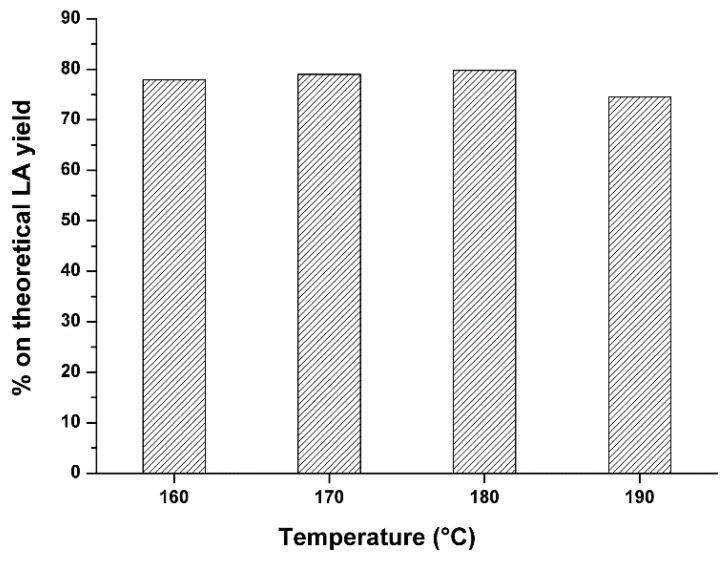
Comparison of the best LA yields by using giant reed as starting biomass, at different hydrolysis temperatures. Agronomic data: “*Pisa*” ecotype; harvest time: august; crop age: 4 years. Reaction conditions: 0.35 g biomass, 5.00 g water, HCl concentration: 1.68 wt %. Hydrolysis time: 15 min.

Maximum theoretical LA yield from hexoses is 71.6 wt % and formic acid makes up the remainder. How close to this theoretical yield is achieved in the conversion process depends on the involved degradation reactions. For an in depth analysis of the obtained LA data, it is useful to express these as percentage on theoretical LA yield, thus taking into account both the overall stoichiometry of the LA reaction and the giant reed cellulose content. Furthermore, this choice is justified by technological and economic reasons which can be very useful for the development of the hydrothermal process on an industrial scale. For the determination of the percentage of the theoretical LA yield, the chemical composition of the starting giant reed sample was evaluated according to the Van Soest method [66]. As example, the conversion of the selected giant reed sample with the following composition, 37.0 wt % of cellulose, 21.0 wt % of hemicellulose and 8.4 wt % of lignin gave the results, in terms of percentage on theoretical LA yield *vs.* temperature, reported in Figure 3.

Above data show that the LA yield does not significantly increases between 160 and 190 °C, approaching about 80% with respect to the maximum theoretical yield. Even at the lowest reported temperature, 160 °C, the obtained LA yield is very high, close to those obtained by the industrial scale-based Biofine Process, which adopts two continuous (plug-flow and back mix) reactors to minimize the reaction time and therefore degradation products/tar formation [67,68,69].

**Figure 4:**
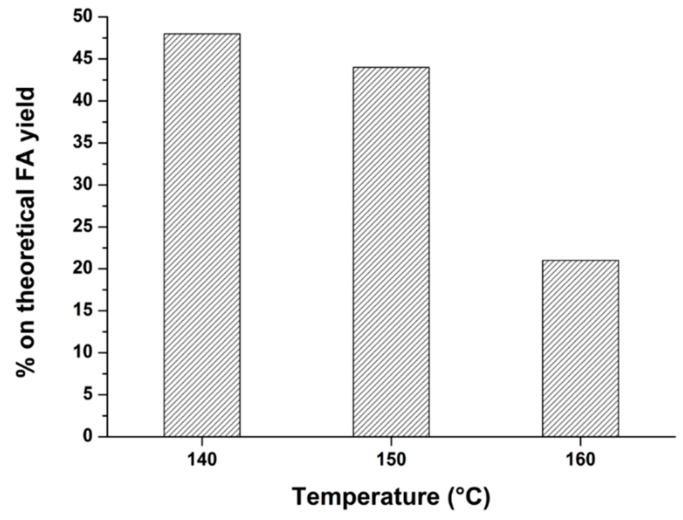
Comparison of the FA yields by using giant reed as starting biomass, at different hydrolysis temperatures. Agronomic data: “*Pisa*” ecotype; harvest time: August; crop age: 4 years. Reaction conditions: 0.35 g biomass, 5.00 g water, HCl concentration: 1.68 wt %. Hydrolysis time: 15 min.

At this level of investigation, after having found the upper temperature limit for LA production, the optimization of the hydrothermal giant reed conversion was focused on that of the first hydrolysis step for FA synthesis, as this furanic compound is particularly sensitive to harsh reaction conditions, as previously stated. In order to find the optimal temperature for FA synthesis, the best FA data reported above were reprocessed as percentage on the theoretical FA yield, thus including both the overall stoichiometry of the FA reaction and the giant reed hemicellulose content. In Figure 4, the comparison of the best FA yields obtained at different temperatures for the hydrothermal conversion of giant reed is reported:

The above figure shows that FA production can be definitely optimized at 140 °C, keeping constant at the same time the remaining optimized reaction conditions. Also in this case, our data are in agreement with the best ones obtained by the Biofine process [69]. It was definitely possible to maximize the LA and FA yield by the MW-approach up to about 80% and 50% (based on the theoretical maximum value and on cellulose/hemicellulose content), respectively. The optimized LA yield is really interesting, especially if compared with that reported in a previous work for the same kind of biomass, which amounted to about 16 wt % [44]. It is noteworthy to underline that the employment of MW irradiation can accelerate the investigation and improve the achieved results while carrying out only a few selected experiments, thus limiting the efforts. Definitely, a proper optimization of the process by the microwave approach has resulted in high FA and LA yields starting from this inexpensive biomass.

**Figure 5:**
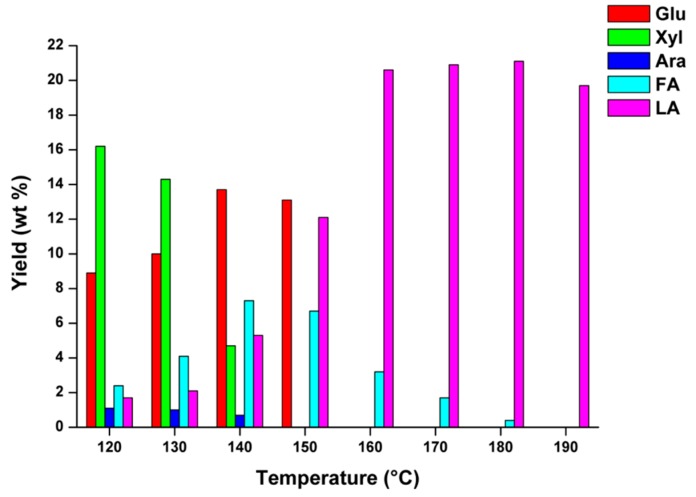
Yield (wt %) in glucose (Glu), xylose (Xyl), arabinose (Ara), furfural (FA) and levulinic acid (LA) as function of the hydrolysis temperature for a giant reed sample. Agronomic data: “*Pisa*” ecotype; harvest time: August; crop age: 4 years. Operating conditions: 0.35 g biomass, 5.00 g water, HCl concentration: 1.68 wt %. Hydrolysis time: 15 min.

Many other hydrolysis tests were performed in order to monitor the concentration of monosaccharaides evolved during giant reed conversion. For this purpose, it was taken into account that the hemicellulose composition of giant reed closely resembles that of the *hardwoods* species, thus showing the prevalence of xylan polysaccharides (xylose represents about 90% of total monosaccharide content) over other non-cellulosic sugars, such as arabinose, galactose, and mannose, which account for the remaining 10% of total monosaccharide content [70]. On this basis, the concentrations of glucose Glu (mainly from C6 sugars and, to a lesser extent, from C5 carbohydrate sugars), xylose Xyl and arabinose Ara were monitored at different reaction temperatures, keeping constant the remaining optimized reaction parameters, thus obtaining a complete profile of the giant reed hydrothermal conversion. Galactose and mannose were not found in the analyzed hydrolysates, because these non-structural carbohydrates were completely degraded as a consequence of the adopted reaction conditions. In Figure 5, the yields in sugar intermediates/products of interest are reported as a function of the hydrolysis temperature for the giant reed biomass.

By choosing hydrolysis temperatures between 120 and 150 °C, it is possible to observe the presence of C5 and C6 sugar monomers, *i.e.*, xylose in the range 120–140 °C (coming from C5 hemicellulose hydrolysis), arabinose in the range 120–140 °C (coming from C5 hemicellulose hydrolysis) and glucose in the range 120–150 °C (coming from cellulose hydrolysis). These results clearly show that the hemicellulose fraction was fully converted below 150 °C and, at this last temperature, no residual pentoses were present. From the point of view of FA recovery, appreciable yields were obtained by using temperatures between 130 and 160 °C, with a maximum yield of about 7.5 wt % at 140 °C. Lower hydrolysis temperatures make incomplete the hemicellulose depolymerization, whilst higher ones promote competitive degradation reactions of the FA in the liquid phase. The complete absence of xylose, arabinose, and glucose at higher hydrolysis temperatures (>140–150 °C) was ascertained, as these monosaccharides can be converted into some desirable intermediates, such as 5-hydroxymethylfurfural (which comes from glucose conversion) [71,72] but also into undesired polymeric insoluble huminic by-products (mainly from condensation of furans, *i.e.*, 5-HMF and/or FA) [73].

**Figure 6:**
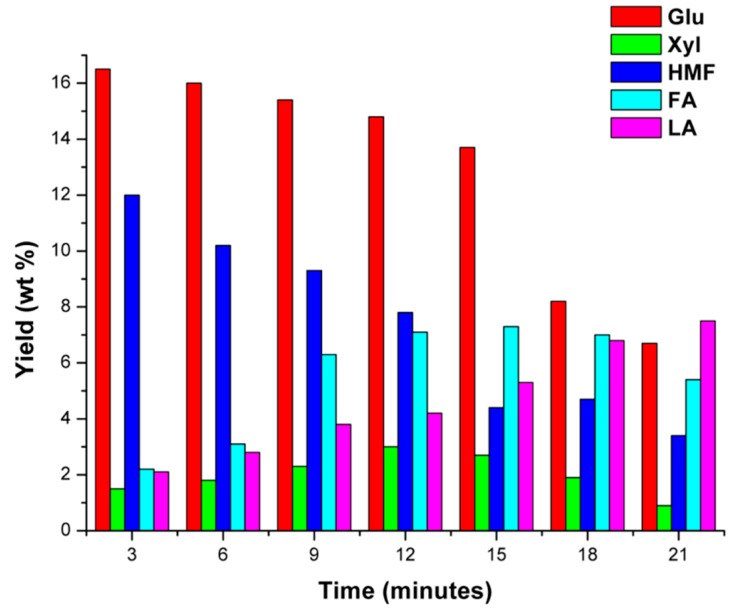
Yield (wt %) in glucose (Glu), 5-hydroxymethylfurfural (5-HMF), xylose (Xyl), furfural (FA) and levulinic acid (LA) as function of the hydrolysis time for a giant reed sample. Agronomic data: “*Pisa*” ecotype; harvest time: August; crop age: 4 years. Operating conditions: 0.35 g biomass, 5.00 g water, HCl concentration: 1.68 wt %. Hydrolysis temperature: 140 °C.

In order to better explain the mechanism for the production of FA and LA in the investigated catalytic process, the reaction temperature of 140 °C was chosen and the kinetic profile of the main intermediates of interest, glucose, xylose, and 5-HMF, together with the desired reaction products was monitored during the reaction progress. The choice of this reaction temperature was strategic, because higher temperatures caused a rapid degradation of the monosaccharides, whilst lower ones were disadvantageous to the 5-HMF production. The results of this kinetic investigation are reported in Figure 6.

The above figure shows that, under the adopted reaction conditions, a low amount of 5-HMF is formed from glucose, this reactive intermediate being readily converted into levulinic acid and humins. Furthermore, longer reaction times favor the degradation of the glucose and xylose, thus not further improving the yield in the intermediates of interest.

Because of the accumulation of organic acids and the liberation of hydrogen ions during the biomass hydrothermal treatment, pH may represent an important parameter to be monitored during the reaction progress. In order to complete the above discussion on the giant reed hydrothermal conversion, the pH was acquired at different reaction temperatures and the obtained data are reported Table 2.

**Table 2:** pH data acquired during the optimized giant reed hydrothermal treatment at different reaction temperatures. Agronomic data: “*Pisa*” ecotype; harvest time: August; crop age: 4 years. Operating conditions: 0.35 g biomass, 5.00 g water, HCl concentration: 1.68 wt %. Hydrolysis time: 15 min.

Temperature (°C)	Initial pH	Final pH	Δ pH
120	0.67	0.41	0.26
130	0.61	0.40	0.21
140	0.62	0.32	0.30
150	0.66	0.32	0.34
160	0.68	0.29	0.39
170	0.67	0.27	0.40
180	0.68	0.26	0.42
190	0.68	0.26	0.42

The obtained data show that the pH is an important parameter for the monitoring of the biomass hydrolysis reaction progress. In fact, the most significant Δ pH variations occurred between 140 and 160 °C, being the most interesting temperature range for the synthesis of the biomass degradation products (Figure 5). However, the Δ pH change is greatest at the higher reaction temperatures, e.g., between 170 and 190 °C, where the obtained LA yields are the highest (Figure 5).

In order to demonstrate the versatility of the hydrothermal process on a larger laboratory scale, the optimized formulation arising from the preliminary microwave investigation was transferred to the autoclave system, with certain interesting consequences from an industrial and practical point of view. Also in this case, the hydrolysis time was further optimized, because of the longer times required by the less efficient heating system. A thermostatic oil bath was used for the autoclave heating and the hydrolysis time was evaluated starting from the autoclave immersion in the oil bath resulting in biomass conversion being carried out under a progressive temperature increase. The transfer of the MW procedure into the autoclave was more difficult for FA, because of the lower reaction times required for its optimal synthesis, due to the degradation of this compound in the liquid phase, which may occur during the transient heating/cooling of the autoclave. The best FA and LA data yields obtained from the giant reed conversion in the autoclave reactor are reported in Table 3, together with those obtained in the control experiment carried out in the absence of HCl catalyst, under the same reaction conditions.

The analysis of the blank experiment confirms that the synthesis of FA and LA needs an efficient acid catalytic system, such as homogeneous hydrochloric acid. Furthermore, the above data show that it is possible to transfer the best results obtained from the microwave reactor to the autoclave system, after an essential optimization of the reaction times. This is due to the slower and less efficient heat transfer of the conventional system, leading to heat dissipation by convection currents, entailing, as a consequence, doubling and tripling of the best hydrolysis times for the synthesis of FA and LA, respectively. Furthermore, the heat transfer by conduction through the walls of the autoclave further limits the efficiency of the heat transfer to the reaction mixture and can also give wall effects caused by inverted temperature gradients, thus leading to the degradation of the solid/liquid suspension directly in contact with the hot surface of the autoclave [74]. These issues are avoided in the MW reactor, since borosilicate glass is almost transparent to microwave irradiation (tan δ = 0.0010), so that coupling of the microwave energy occurs with molecules present in the reaction media.

**Table 3:** Best furfural (FA) and levulinic acid (LA) yields obtained for giant reed hydrothermal conversion, performed in the autoclave reactor, under the previously optimized reaction conditions. Agronomic data: “*Pisa*” ecotype; harvest time: October; crop age: 4 years. Operating conditions for FA synthesis: 2.20 g biomass, 30.00 g water, HCl concentration: 1.68 wt %. Hydrolysis time = 30 min; oil bath temperature = 210 °C; pressure of loaded N_2_ = 30 bar. Operating conditions for LA synthesis: 2.20 g biomass, 30.00 g water, HCl concentration: 1.68 wt %. Hydrolysis time = 60 min; oil bath temperature = 210 °C; pressure of loaded N_2_ = 30 bar.

Catalyst	FA Yield (wt %)	LA Yield (wt %)
HCl	8.9	19.6
No catalyst	0.2	1.1

**Figure 7:**
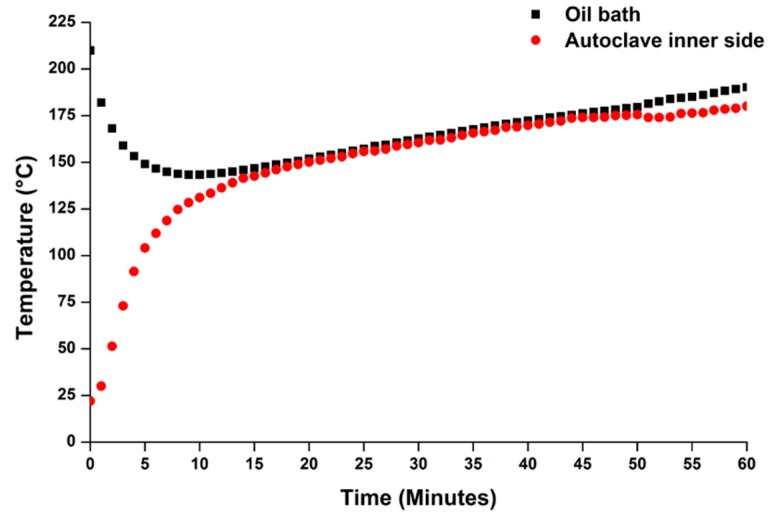
Temperature trend of the autoclave inner side and the oil bath, as a function of hydrolysis time. Reagents and operating conditions: 2.20 g “*Pisa*” ecotype biomass, 30.00 g water, HCl concentration: 1.68 wt %, oil bath temperature = 210 °C, pressure of loaded N_2_ = 30 bar. The hydrolysis reaction was performed in a 150 mL AISI 316 steel autoclave.

The above statements are well justified by monitoring the internal temperature of the autoclave during the hydrothermal process and that of the oil bath, both as a function of time. The temperatures were acquired by means of two separate thermocouples, at regular intervals of one minute between each measurement and the next one. The experimental data of a giant reed hydrolysis experiment are reported in Figure 7.

Oil bath temperature collapses from 210 °C to about 140 °C during the first 10 min because of the heat exchange between the oil bath, the autoclave, and the external environment (by conduction/convection). From the point of view of the reaction mixture, it takes about 15 min to reach 140–150 °C, which represents the optimal range for FA synthesis (on the basis of the MW screening experiments, as reported in Figure 1 and Figure 5), and about 30 min to reach 160–170 °C, which was considered optimal for LA production (on the basis of the MW screening experiments, as reported in Figure 1 and Figure 5). These long heating times required for the autoclave system are avoided, or anyway minimized by the MW approach (from 5 to 10 min for heating the reaction mixture up to the set-point temperature for FA and LA synthesis, respectively), thus further confirming the greater efficiency of the MW system. In this kind of study, temperature monitoring/control was definitely fundamental for transfer of the reaction route from one system to the other one.

### 2.2. Optimization of FA and LA Production as Function of the Harvest Time

At this level of investigation, only the chemical conversion was considered and optimized, taking into account that giant reed samples have a chemical and botanical composition. However, by using the same optimized reaction conditions but with biomass samples with different agronomic characteristics, it was possible to evidence some interesting and recurrent differences in FA and LA yields. For example, giant reed suitability for FA production changes in relation to the crop ecotype (e.g., ecotype “*Torviscosa*” selected in Northern Italy seems better than “*Pisa*” ecotype selected in Central Italy), but the biomass choice should take into account also distinctive agronomic parameters, such as the harvest time and the fertilization rate, strongly affecting crop yield and biomass quality. For hydrothermal exploitation, biomass should have the highest possible content of hemicellulose/cellulose and the lowest one of lignin. The content of these macro constituents strongly depends on the above agronomic variables. Ultimately, these agronomic parameters may give a strong contribution to the overall optimization of the biomass conversion process and have to be considered for a scale up of the process on a larger scale.

**Figure 8:**
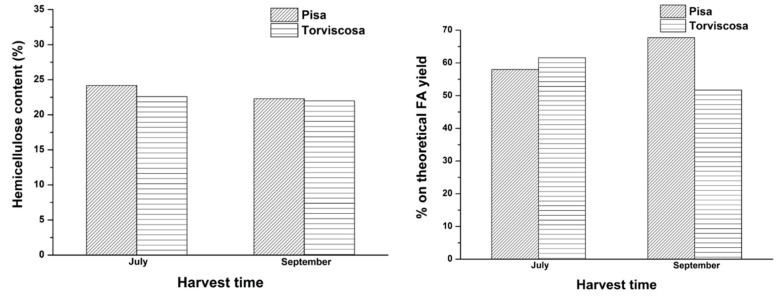
Hemicellulose content and percentage on theoretical FA yield, both as a function of the harvest time of two giant reed ecotypes. Agronomic data: crop age of “*Pisa*” ecotype: 4 years; crop age of “*Torviscosa*” ecotype: 3 years. Reaction conditions: 0.35 g biomass, 5.00 g water, HCl concentration: 1.68 wt %. Hydrolysis time: 15 min.

On this basis, many hydrolysis tests were carried out to investigate the dependence of FA and LA yield on the harvest time of giant reed. In Figure 8, hemicellulose content and percentage on theoretical FA yield, both as a function of the harvest time of “*Pisa*” and “*Torviscosa*” ecotypes, are reported.

**Figure 9:**
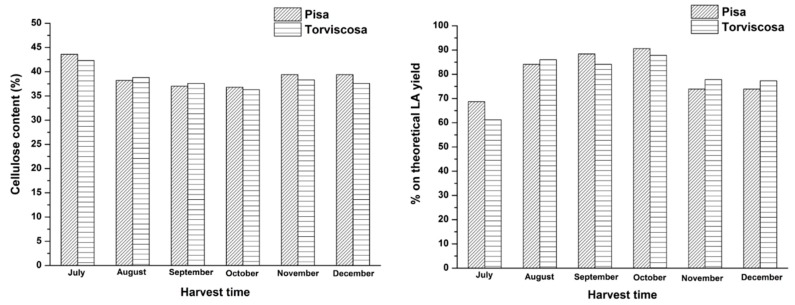
Cellulose content and percentage on theoretical LA yield, both as a function of the harvest time of two giant reed ecotypes. Agronomic data: crop age of “*Pisa*” ecotype: 4 years; crop age of “*Torviscosa*” ecotype: 3 years. Reaction conditions: 0.35 g biomass, 5.00 g water, HCl concentration: 1.68 wt %. Hydrolysis time: 15 min.

Instead, in Figure 9, cellulose content and percentage on theoretical LA yield, both as a function of the harvest time of the two giant reed ecotypes, are reported.

The above figures show that the two giant reed ecotypes have a similar content of hemicellulose/cellulose and similar FA/LA yields, even at different harvest times. From a purely chemical point of view, some slight differences can be highlighted, such as that regarding the percentage on the theoretical LA yield of both ecotypes, which increases going from July up to August/September and then tends to decrease (Figure 9). However, this attained advantage is not significant and real, because giant reed maintains a high productivity (or yield in dry matter, t·ha^−1^) during the entire year. With this perspective, “*Pisa*” ecotype shows much higher dry matter yield values than “*Torviscosa*” ecotype (varying in the average range 22–34 t·ha^−1^ *vs.* 12–20 t·ha^−1^, respectively, during the investigated harvest time). Therefore, by taking into account the most promising *“Pisa”* ecotype, its productivity increases beginning from October over the next months (from about 25 up to 34 t·ha^−1^, respectively) and this effect offsets the slightly lower LA yields of the last months (Figure 9). Figure 8 and Figure 9 definitely show that by balancing the chemical and agronomical parameters, it is possible to further improve FA and LA yields to up to 70% and 90% on the theoretical yield, respectively. The high productivity of giant reed might be increased by coupling the early cuts with the corresponding regrowth (double harvest), thus further improving the exploitation to LA within the biorefinery. These results highlight the versatility of this biomass towards different conversion approaches, as also recently evidenced by Ragaglini *et al.* [45] in an independent biochemical study on the effect of the harvest time and frequency on the biomethane yield potential of giant reed. In addition to the above mentioned agronomical advantages of giant reed, it is useful to highlight that its production cost is still low (about 25 €·ton^−1^ of dry matter) with respect to annual herbaceous biomasses such as sorghum and therefore its exploitation as starting biomass for chemical transformations is further confirmed.

### 2.3. Yield in Solid Residue: Recovery and Exploitation Possibilities

**Figure 10:**
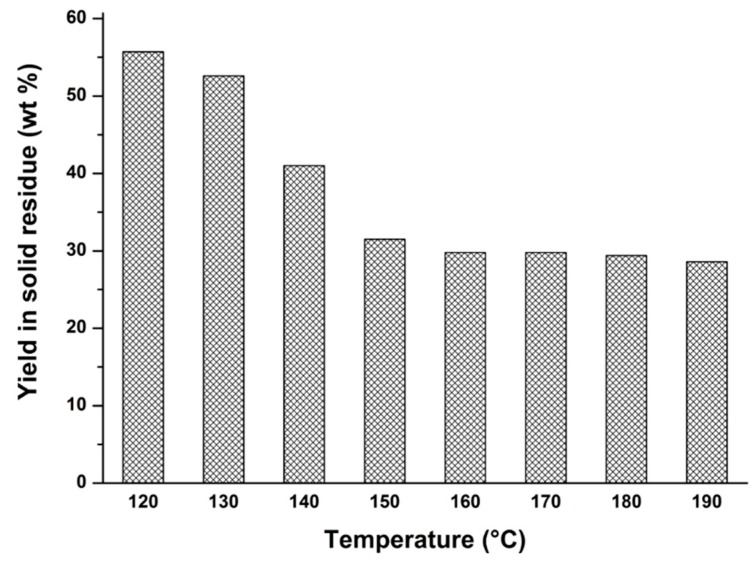
Yield of solid residue (wt %) as a function of the hydrolysis temperature for a giant reed biomass. Operating conditions: 1.63 g biomass, 23.33 g water, HCl concentration: 1.68 wt %. Hydrolysis time: 15 min.

After LA recovery in the second hydrolysis step, it is necessary to recover the final solid residue of the hydrolysis reaction in order to complete the integral biomass exploitation. At the appropriate temperature for LA formation, all sugar monomers have been converted into desirable and undesirable products, as previously stated. Therefore, it is not possible to further optimize the hydrothermal process and attention can be focused on the solid residue recovered at the end of the overall reaction, which includes polymeric humic products (from 5-HMF and FA fail reactions) and the lignin fraction, which has been degraded in various ways. Because the lignin fraction of giant reed is abundant (~20–25 wt %), it should be interesting to recover and exploit the solid “*lignin-like*” residue downstream of the hydrothermal process. Furthermore, the recovery of the hydrolysis residue at different hydrolysis temperatures should allow us to obtain interesting information about the biomass liquefaction during the hydrothermal conversion, thus justifying the overall mass balance of the intermediates/products. In Figure 10, the yields of solid residue recovered at the end of the optimized hydrothermal giant reed conversion are reported at different hydrolysis temperatures.

The above figure provides evidence that the yield of solid residue has been significantly reduced between 120 and 150 °C, this being the temperature range useful for hemicellulose and cellulose depolymerization. Therefore, this parameter is important to monitor the hydrolysis reaction, thus justifying and supplementing data yields coming from the optimization of the hydrolysis reaction. Regarding the carbon balance, in addition to the previously mentioned compounds (glucose, xylose, arabinose, furfural, and levulinic acid), it is necessary to consider also formic acid which decomposes at high temperature. On the basis of the stoichiometry of the reactions, formic and levulinic acids should be produced in a molar ratio of 1:1 but in most cases their determined amounts (on a molar basis) are different. At low temperature (120 °C) the carbon balance is almost complete, probably due to the low presence of both formic and levulinic acids and of different by-products. On the other hand, when the reaction temperature increases (higher than 140 °C) the carbon balance fails by about 30 wt %. This lacking percentage could be ascribable to the presence of solubilized humins, to the small amount of soluble phenols (monomers, dimers, and oligomers) deriving from soluble lignin, but mainly due to the significant portion of non-condensable products resulting from the degradation of all the mixture compounds, as the well-known formic acid degradation. In the MW reactor it was not possible to analyze this fraction and a parallel investigation in order to collect and characterize these by-products from the autoclave system is now in progress. Ultimately, after LA recovery, an abundant waste fraction is recovered as solid, amounting to ~30 wt % of the weight of the starting biomass, which includes both degraded/condensed lignin units and furanic humins of C5 and C6 carbohydrate source. A detailed characterization of this charred material has been discussed in recent works [75,76], and some new, green and low cost application perspectives have been proposed. In detail, humins, incorporated into the final solid residue, coming from FA degradation and also 5-HMF condensation products deriving from C6 sugar dehydration, could be exploited together with the residual lignin fraction, thanks to their residual free hydroxyl and carbonyl groups, making the solid residue once again a reactive material. An in depth study in order to ascertain the influence of the humin amount on the carbon balance is now in progress.

The investigated hydrothermal process is mainly based on hemicellulose and cellulose fractionation to give FA and LA, respectively, and these platform chemicals compensate the costs of the entire process and generate profit. The exploitation of the *Arundo donax* L. hydrolysis residue, hitherto considered as a waste fraction of the hydrothermal process, allows the completion of the giant reed biorefinery cycle, adding at the same time further profit and minimizing the environmental pollution and waste management.

## 3. Experimental Section

### 3.1. Field Experiment and Sample Preparation

Giant reed samples were provided by the Institute of Life Sciences “*Scuola Superiore Sant’Anna*” of Pisa. The two giant reed ecotypes (Pisa and Torviscosa) had been cultivated since April 2006 in Central Italy at the Enrico Avanzi Interdepartmental Centre for Agro-Environmental Research (CIRAA) of the University of Pisa, located in San Piero a Grado (PI) (latitude 43°68’N, longitude 10°35’E). The soil was representative of the lower Arno River plain and characterized by a shallow water table, with a maximum depth of 120 cm during the dry season, and by a good supply of macro-elements. The crops were managed under optimal growth conditions suitable for the Mediterranean environment. No crop diseases and irrigation were detected during the experimental period. Regarding the agronomic investigation on the effects of harvest time, in the 2009 growing season, monthly samplings were carried out on 3 year-old crops, from July to November. At each harvest time, biomass fresh weight was determined by sampling a 2 m^2^ area within 4 different plots (12 × 3 m each). After, the whole above-ground biomass was dried at 65 °C until constant weight, in order to assess the dry biomass yield. Subsequently, biomass samples for chemical analyses were prepared by milling in a Retsch SM1 rotor mill equipped with a 1 mm grid.

### 3.2. Cellulose, Hemicellulose, and Lignin Determination

Cellulose, hemicellulose, and lignin analytical determinations in the biomass were performed at the Department of Agriculture Food and Environment of the University of Pisa, according to the Van Soest method, which employs NDF, ADF, and ADL determination. Cellulose percentage was calculated from acid detergent fiber (ADF) and lignin (ADL) (ADF minus ADL), whereas hemicellulose percentage was calculated from neutral detergent fiber (NDF) and ADF (NDF minus ADF). Finally, lignin represented the solid residue obtained after the acid hydrolysis of the ADF residue, and it was expressed as Acid Detergent Lignin (ADL) [66]. The analysis was carried out in triplicate and the reproducibility was within 3%.

### 3.3. Hydrolysis Experiments

The optimization of the hydrolysis reaction of the investigated herbaceous biomasses was carried out by using a commercially available *mono-mode* microwave reactor (CEM Discover S-Class System, NC, USA) [77,78], in a 10 or 35 mL vessel, containing a teflon stir bar. This instrument was equipped with a built-in keypad for programming the reaction procedures and allowing *on-the-fly* changes. The output power was maximized to continuously deliver 300 W (power could be set between 0 and 300 W), sufficient for rapid heating of the aqueous slurry. Temperature measurement during the reaction was achieved by an IR sensor positioned at the bottom of the cavity, below the vessel. The reactor was closed and irradiated up to the set-point temperature, by employing a fixed ramping time, thus carrying out reproducible experiments. No air cooling during the MW irradiation (cooling system “*PowerMAX*”) was used. At the end of each experiment, the reactor was rapidly cooled up to room temperature by means of air which was blown directly on the surface of the reactor and a small quantity of the obtained solid/liquid suspension was filtered on a 0.2 μm Whatman filter.

Instead, a 150 mL AISI 316 steel autoclave was adopted for traditional hydrolysis reactions. Inside the autoclave, a teflon vessel was employed to minimize corrosion problems. It was equipped with a loading access with a Whitey tap for nitrogen loading and for discharge of the reaction product by siphoning. Furthermore, the autoclave was equipped with an access for inserting a thermocouple for the evaluation of the internal temperature of the reactor during the hydrolysis reaction. The biomass and other reagents were loaded into the reactor together with a Teflon stir bar. The autoclave was closed and pressurized with nitrogen at 30 bar and then introduced into an oil bath, which was previously thermostated at the set-point temperature (210 °C). The hydrolysis time was evaluated starting from the autoclave immersion in the oil bath. The heating of the oil bath was performed by using a VWR Magnetic hot plate stirrer model VMS-C10-2 (28 × 28 cm), equipped with a VWR electronic contact thermometer model VT-5 for the oil bath temperature control. At the end of the hydrolysis reaction, the reactor was cooled to room temperature, then degassed and finally a small quantity of the obtained suspension was filtered with a 0.2 μm Whatman filter.

The filtrate solution was properly diluted with water and analyzed by means of UV-Vis spectroscopy (Jasco, Easton, PA, USA) and GC chromatography (Perkin Elmer, Waltham, MA, USA for furfural and levulinic acid determination, respectively. Furthermore, the simultaneous determination of xylose, glucose, arabinose, furfural, and levulinic acid was carried out by HPLC chromatography (Perkin Elmer, Waltham, MA, USA). At the end of each hydrolysis experiment, the solid residue was washed with water up to neutrality and dried at 105 °C in a thermo-ventilated oven (Thermo Scientific, Waltham, MA, USA), to constant weight. Giant reed hydrolysis experiments were carried out in duplicate.

### 3.4. Identification of FA and LA in the Hydrolyzate by GC-MS

The starting aqueous solution was extracted with methyl isobutyl ketone and the diluted extract (about 0.1–0.2 μL) was qualitatively analyzed by gas chromatography/mass spectrometry (GC-MS) (Hewlett-Packard HP, Palo Alto, CA, USA). For the analysis, a gas-chromatograph Hewlett-Packard HP 6890 equipped with a MSDHP 5973 detector and with a G.C. column Phenomenex Zebron with a 100% methyl polysiloxane stationary phase (column length 30 m, inner diameter 0.25 mm and thickness of the stationary phase 0.25 μm) was used. The transport gas was helium 5.5 and the flow was 1 mL/min. The temperature of the injection port was set at 250 °C, carrier pressure at 100 kPa and split flow at 3.40 ms^−1^. The oven was heated at 30 °C for 5 min, and then the temperature was raised at 5 °C/min up to 250 °C.

### 3.5. Determination of Furfural by UV-Vis Spectroscopy

For FA determination, a double beam UV-Vis spectrophotometer JASCO V-530 was adopted. The absorbance at 280 nm was chosen for FA determination. The analysis of the hydrolyzed liquid fraction was performed after the acquisition of the calibration curve absorbance/concentration of standard solutions. The analysis was carried out in triplicate and the reproducibility was within 3%.

The FA yield with respect to the weight of the raw biomass was calculated as follows:

(1) FA Yield (wt %) = [FA recovered after reaction (g)/dried biomass (g)] × 100

### 3.6. Determination of Levulinic Acid by GC Chromatography

The determination of LA was performed by a Perkin Elmer Autosystem XL gas chromatograph (Waltham, MA, USA), which was equipped with a flame ionizator detector (FID). The adopted capillary column contained a stationary phase Zebron ZB-WAX 100% polyethylene glycol stationary phase (30 m × 0.32 mm × 0.50 μm). Helium was used as carrier gas. The following temperature program was adopted: initial temperature of 55 °C for 15 min; heating rate of 10 °C/min; final temperature of 200 °C for 10 min. The GC analysis of levulinic acid was performed by using THF as internal standard. The analysis was carried out in triplicate and the reproducibility was within 3%.

The LA yield with respect of the weight of the raw biomass was calculated as follows:

(2) LA Yield (wt %) = [LA recovered after reaction (g)/dried biomass (g)] × 100

### 3.7. Simultaneous Determination of Glucose, Xylose, Arabinose, Furfural. and Levulinic Acid by Means of HPLC

The quantitative determination of glucose, xylose, arabinose, furfural, and levulinic acid was performed by High Performance Liquid Chromatography (HPLC). For this purpose, a Perkin Elmer Flexar Isocratic Platform, which was equipped with a differential refractive index detector, was used. 20 μL samples were loaded into a Polypore CA column (4.6 mm × 220 mm × 10 μm) and eluted with 0.5 mM H_2_SO_4_ at a flow rate of 0.1 mL/min. The column was maintained at 60 °C, and the calibration was carried out using a commercial standard of FA. At least three replicates for each concentration of FA and LA standard were carried out. The reproducibility of the technique was within 2%.

The yield of product with respect to the weight of the raw biomass was calculated as follows:

(3) Yield (wt %) = [Product recovered after reaction (g)/dried biomass (g)] × 100

The yield of FA based on theoretical yield was calculated as follows:

(4) FA Yield based on theoretical yield (%) = [FA recovered after reaction (g)/(dried biomass (g) × hemicellulose content × 0.7273)] × 100

The yield of LA based on theoretical yield was calculated as follows:

(5) LA Yield based on theoretical yield (%) = [LA recovered after reaction (g)/(dried biomass (g) × cellulose content × 0.7155)] × 100

Finally, the yield of solid residue was calculated as follows:

(6) Yield of solid residue (wt %) = [Solid recovered after hydrolysis reaction (g)/dried starting biomass (g)] × 100

### 3.8. pH Monitoring during the Giant Reed Hydrothermal Conversion

The pH measurements of the starting slurries and the final hydrolyzed solutions were acquired using an OMEGA^®^ PHH-SD1 pH meter.

## 4. Conclusions

The acid-catalyzed hydrothermal conversion of giant reed was investigated and optimized. This biomass was chosen because of its suitability as a biomass crop in the Mediterranean area, also under extreme growing conditions. Water was adopted as reaction medium, thus ensuring a more green and sustainable approach to the entire process. From the results shown in this work, microwaves were shown to represent a very efficient alternative to the traditional heating route to give FA and LA. In fact, microwave heating allowed a fast screening of the best hydrolysis conditions, thus allowing remarkable energy and time savings and ensuring good reproducibility between all experiments. Dilute hydrochloric acid was found to be a very effective acid catalyst for the investigated hydrolysis reaction. By using the diluted acid approach, it was found that the hydrolysis temperature and time were the main reaction variables to be carefully optimized: in detail, FA formation needed milder reaction conditions, whilst LA required more severe ones. It is also interesting to highlight that the best optimized FA and LA yields achieved in this study employing MW irradiation were very high, when compared with the data reported in the literature. Furthermore, it was found that harvest time was not a discriminating parameter for the further optimization of both FA and LA production, thanks to the very high productivity of the giant reed throughout the year. Finally, another important goal achieved from the present investigation represents a real possibility of transferring the MW procedure to the autoclave from the standpoint of scale-up, underlining the techno-economic feasibility of employing this biomass also on the industrial scale. On the basis of the obtained results, this biomass represents a very promising starting material for the contemporary FA and LA production, and it must surely be taken into account as a potential starting feedstock, within a biorefinery plant in the Mediterranean basin.

## Supplementary Materials

Supplementary materials can be accessed at: http://www.mdpi.com/1420-3049/20/12/19760/s1.
