# Supplementary material for: Hydrothermal Conversion of Giant Reed to Furfural and Levulinic Acid: Optimization of the Process under Microwave Irradiation and Investigation of Distinctive Agronomic Parameters
Source: Molecules. 2015 Nov 30;20(12):21232–53. doi: 10.3390/molecules201219760 (PMC6332085; doi:10.3390/molecules201219760)
Supplement: Supplementary file 1 [file molecules-20-19760-s001.pdf]

Supplementary Materials: Hydrothermal Conversion of Giant Reed to Furfural and Levulinic Acid: Optimization of the Process under Microwave Irradiation and Investigation of Distinctive Agronomic Parameters

Claudia Antonetti, Enrico Bonari, Domenico Licursi, Nicoletta Nassi o Di Nasso and Anna Maria Raspolli Galletti

At the beginning of the present study, in order to identify the appropriate reaction temperatures for the FA and LA synthesis, a wide screening of the reaction temperatures was investigated (130–190 °C) and the obtained hydrolyzates were analyzed by GC-MS. As example, the GC-MS chromatograms of the reactions at 160 and 190 °C are reported in Figures S1 and S2.

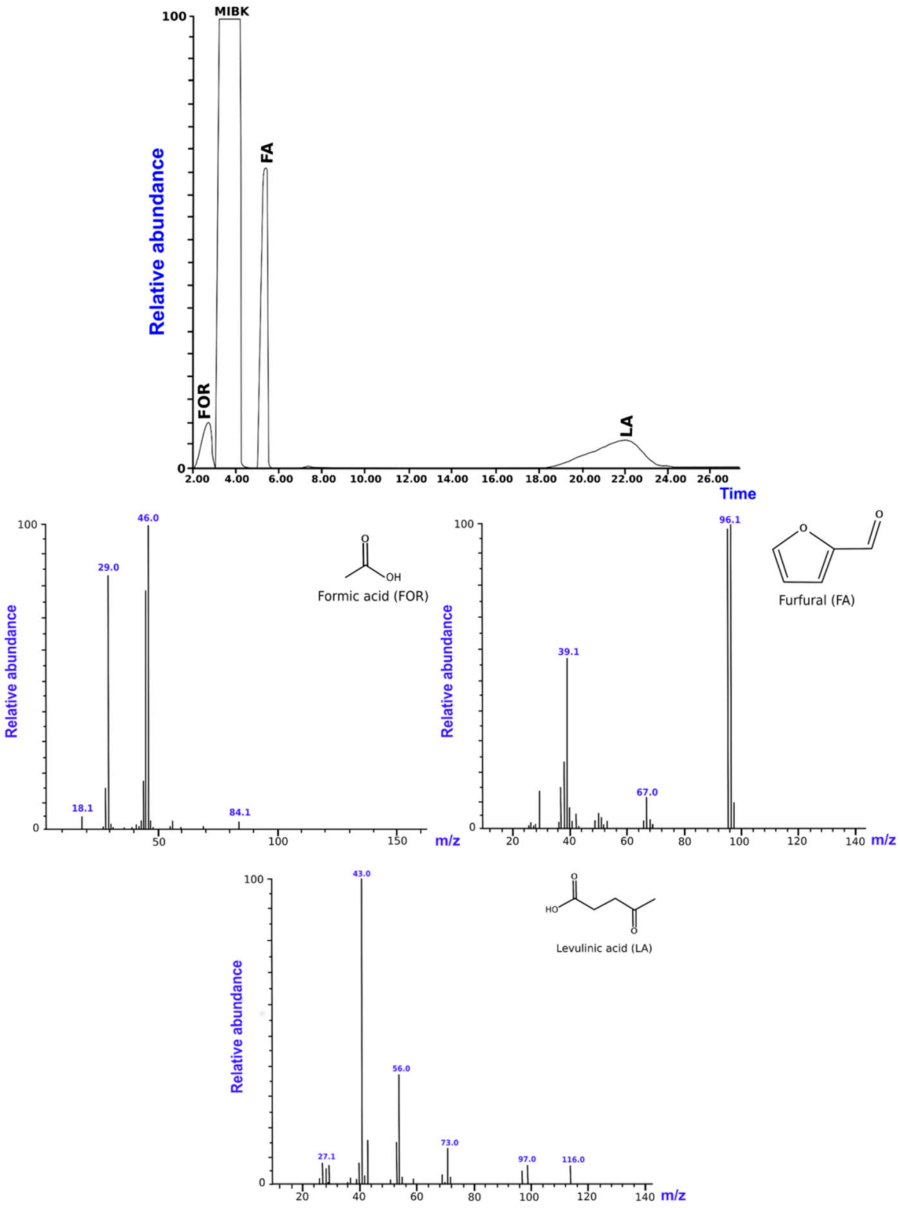

Figure S1. GC-MS analysis of reactions at 160 °C. **Agronomic data**: "*Pisa*" ecotype; harvest time: August; crop age: 4 years. **Operating conditions**: 0.35 g biomass, 5.00 g water, HCl concentration: 1.68 wt %. Hydrolysis time: 15 min.

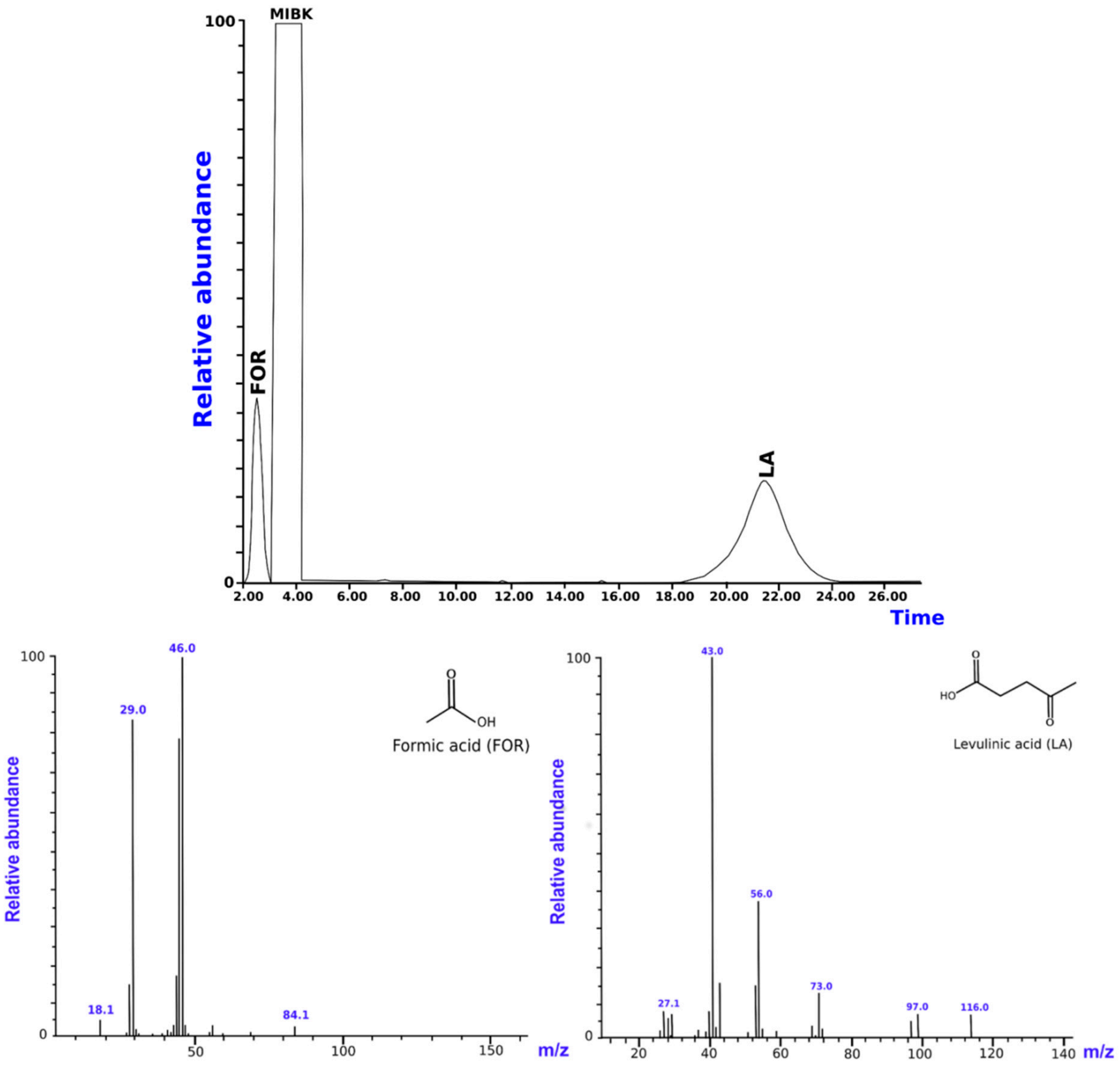

Figure S2. GC-MS analysis of reactions at 190 °C. **Agronomic data**: "*Pisa*" ecotype; harvest time: August; crop age: 4 years. **Operating conditions**: 0.35 g biomass, 5.00 g water, HCl concentration: 1.68 wt %. Hydrolysis time: 15 min.
